# Factors influencing community pharmacists’ provision of health promotion and disease prevention services in Jordan: Qualitative thematic analysis and deductive mapping to the COM-B model

**DOI:** 10.1371/journal.pone.0355315

**Published:** 2026-08-05

**Authors:** Ameerah Hasan Ibrahim, Ibtihal Ibrahim, Tahani Alwidyan, May Tayyem

**Affiliations:** 1 Department of Pharmacy, Faculty of Pharmacy, Al-Zaytoonah University of Jordan, Amman, Jordan; 2 Department of Clinical Pharmacy, Faculty of Pharmacy, Jordan University of Science and Technology, Irbid, Jordan; 3 Department of Clinical Pharmacy and Pharmacy Practice, Faculty of Pharmaceutical Sciences, The Hashemite University, Zarqa, Jordan; 4 Department of Pharmaceutical Technology and Cosmetics, Faculty of Pharmacy, Middle East University, Amman, Jordan; PLOS ONE, UNITED KINGDOM OF GREAT BRITAIN AND NORTHERN IRELAND

## Abstract

**Background:**

Non-communicable diseases represent a major cause of morbidity and mortality in Jordan and place increasing demands on primary healthcare services. Community pharmacists (CPs) play an important role in providing health promotion and disease prevention (HPDP) services. However, limited evidence exists regarding factors influencing their involvement in these activities.

**Objective:**

The study aimed to explore CPs’ perceptions and experiences regarding the provision of HPDP services in Jordan and to identify factors influencing service delivery, including facilitators and barriers.

**Methods:**

Between January and April 2025, face-to-face semi-structured interviews were conducted with CPs in Amman, Jordan, recruited through purposive and snowball sampling. Interviews were audio-recorded, transcribed verbatim, and analyzed in NVivo® QSR 14 using inductive thematic analysis following Braun and Clarke’s six-step approach. Transcripts were independently coded by two researchers. The identified themes were subsequently deductively mapped to the Capability, Opportunity, Motivation-Behavior (COM-B) framework. Data collection and analysis were conducted concurrently, and data saturation was achieved when no new themes emerged.

**Results:**

Fifteen CPs participated. Four themes were identified: (1) Diverse and evolving roles of CPs in HPDP; (2) Barriers to providing HPDP services, including workload, limited patient engagement, lack of appreciation from pharmacy owners, and the absence of financial incentives; (3) Professional requirements and capacity building, including continuing professional development, undergraduate training, and physician collaboration; and (4) Impact of providing HPDP services on patients, pharmacists, and the healthcare system. Deductive mapping indicated that pharmacists’ engagement in HPDP services was influenced by capability-, opportunity-, and motivation-related factors.

**Conclusions:**

CPs in Jordan play an active role in HPDP services; however, their contribution is hindered by structural, educational, and motivational barriers. Addressing these barriers through targeted training, infrastructure development, enhanced pharmacist-physician collaboration, and public awareness initiatives is essential to strengthen pharmacist-led HPDP services and support their integration into broader public health strategies in Jordan.

## Introduction

Non-communicable diseases, such as diabetes, hypertension, and cardiovascular disease, are leading causes of morbidity and mortality worldwide [1]. Addressing this growing burden requires effective health promotion and disease prevention (HPDP) strategies. Health promotion has been defined by the World Health Organization (WHO) as enabling people to live healthier lives by increasing their control over their health through improved health literacy and multisectoral actions to produce healthy environments and behaviors [2]. Accordingly, disease prevention involves population-level and individual-level interventions aimed at reducing the incidence, progression, and complications of chronic diseases and other health conditions [2].

HPDP services are fundamental components of public health efforts to improve population wellbeing. These services involve several interventions, such as screening programs, health education initiatives, and lifestyle counseling [3]. Available evidence indicates that HPDP services contribute to increased productivity, reduced disease burden, and lower healthcare costs across diverse populations [4–7]. While HPDP services are widely recognized as valuable, their integration into routine healthcare practice remains challenging. Barriers include limited time, lack of adequate training, poor integration of information technology systems, and limited professional experience among healthcare providers (HCPs) [8,9].

Worldwide, community pharmacists (CPs) are increasingly recognized as highly accessible HCPs, particularly in low- and middle-income countries [10–12]. As the scope of pharmacy practice continues to expand, CPs are anticipated to deliver preventive services in addition to their traditional dispensing responsibilities [12–14]. This shift is largely driven by the growing prevalence of chronic disease [15], an aging population [15], increasing pressure on healthcare systems [15], and greater public awareness of health risks such as obesity [16] and tobacco smoking [17]. Consequently, CPs are increasingly expected to contribute to public health initiatives through the delivery of HPDP services [10]. However, the extent to which pharmacists engage in these activities may be influenced by individual, organizational, and system-level factors [18].

Several studies have shown that pharmacist-led interventions are effective across several important areas such as smoking cessation [14], diabetes care [19], hypertension management [19], dyslipidemia management [19], immunization uptake [20], contraception counseling [21], and osteoporosis prevention [22]. Moreover, during the COVID-19 pandemic, CPs played a vital role by providing point-of-care testing, telepharmacy services, and mass vaccination programs, highlighting their contribution to enhancing healthcare reach and system resilience [23].

In Jordan, CPs have taken on a more active role in public health initiatives, particularly through telepharmacy services and COVID-19 vaccination programs [24]. However, recent studies identified substantial training and infrastructure needs among CPs [24–26]. Additionally, qualitative research examining the role of Jordanian CPs in mental health support has identified communication difficulties and the need for better training in sensitive, high-stigma healthcare areas [27]. These findings point to continuing structural and professional challenges that may hinder efforts to expand CPs’ public health role.

Research investigating the role of CPs in providing HPDP services in Jordan remains limited. Few studies have explored CPs’ experiences of delivering HPDP services in routine practice, their perceived roles, and the contextual factors influencing service delivery within community pharmacy settings [28–30]. Furthermore, most existing studies have employed quantitative approaches, providing limited insight into pharmacists’ experiences and the context in which HPDP services are delivered in everyday practice [28–30]. Existing quantitative evidence suggests that HPDP services are not fully integrated into routine practice in Jordan due to systemic constraints [28–30]. This gap limits the ability to fully utilize CPs’ potential in advancing public health.

Understanding the factors that influence CPs’ engagement in HPDP services requires a theoretically informed perspective on the professional behaviors involved in service delivery [31]. The Capability, Opportunity, Motivation-Behavior (COM-B) framework proposes that behavior is influenced by an individual’s capability, opportunity, and motivation [31]. As the provision of HPDP services represents a professional behavior shaped by individual, organizational, and system-level factors, the COM-B framework provides a useful theoretical lens for interpreting factors that may influence pharmacists’ engagement in these activities [31].

Therefore, this study sought to explore CPs’ perceptions and experiences regarding the provision of HPDP services in Jordan and to identify factors influencing service delivery, including barriers and facilitators. Gaining a deeper understanding of these aspects is crucial for guiding policy decisions, enhancing pharmacy education, and promoting the effective integration of CPs into Jordan’s broader public health system.

## Methods

### Study design

A qualitative study employing semi-structured interviews was conducted to explore CPs’ perspectives on providing HPDP services in Jordan. The study was exploratory in nature and aimed to gain an in-depth understanding of CPs’ experiences and perceptions regarding the provision of HPDP services. This study adopted an applied qualitative descriptive approach to generate a practice-oriented account of CPs’ experiences and perceptions regarding HPDP service provision. It was conceptually informed by the COM-B framework. Thematic analysis was conducted inductively, and the COM-B framework was subsequently used to provide a theoretical interpretation of the identified themes through deductive mapping. This study was reported in accordance with the Consolidated Criteria for Reporting Qualitative Research (COREQ) checklist (see S1 File) [32].

### Participants and setting

Eligible participants were CPs registered with the Jordan Pharmacists Association, who had at least one year of experience working in a community pharmacy setting in Amman, Jordan, and expressed willingness to participate. The interviews were conducted face-to-face at the community pharmacies.

### Sampling and recruitment

Purposive and snowball sampling were used to recruit participants between 1 January 2025 and 30 April 2025 to ensure diversity in experiences and perspectives regarding HPDP service provision. Purposive sampling was used as the primary recruitment strategy to capture variation in participants’ demographic and professional characteristics, while snowball sampling facilitated access to additional eligible participants. Pharmacists were identified through the official Jordan Pharmacists Association Directory [33] and contacted using publicly available telephone numbers. Potential participants were provided with an information sheet outlining the study objectives, procedures, and voluntary nature of participation. Written informed consent was obtained from all participants prior to data collection. During the interviews, participating CPs were asked to nominate and suggest the name of a potential CP participant.

### Data collection

Semi-structured interviews were conducted by a trained female interviewer, Ibtihal Ibrahim (II). The interviewer (II) holds a Master’s degree in Clinical Pharmacy, is a qualified PharmD, and was a lecturer at Al-Zaytoonah University of Jordan at the time of the study. No participants were known to the interviewer prior to the study commencement. Furthermore, the interviewer did not practice in community pharmacy. Additionally, participants were aware that the researcher (II) worked as a lecturer at Al-Zaytoonah University of Jordan and were given an overview of the project. The researcher (II) also had an interest in the research topic of assessing CPs’ perceptions regarding the provision of HPDP services in Jordan. To minimize the potential influence of researcher assumptions on data collection and interpretation, reflexivity was maintained throughout the study. The interviewer recorded field notes and documented reflections and observations following interviews to capture contextual information relevant to data interpretation. Regular discussions were held within the research team to critically examine emerging interpretations and potential sources of bias.

An interview topic guide with prompts (Table 1) was developed based on a review of relevant literature and expert input. The topic guide was pilot-tested with two CPs who were not included in the final analysis, and refined accordingly. During the interviews, probing questions were used to clarify participants’ responses, explore emerging issues in greater depth, and encourage participants to elaborate on their experiences and perspectives regarding HPDP services.

**Table 1:** Interview topic guide and prompts.

**1. Could you tell me about the roles /activities of community pharmacists in Jordan?** • For example, dispensing of drugs, patient counseling, providing pharmaceutical care, identifying health issues, and making appropriate evidence-based product recommendations **2. In your opinion, what do the terms health promotion and disease prevention mean to you?** • Differences, similarities **3.** I would now like to show you a card with the definitions of health promotion and disease prevention. Can I ask you to read this please? **Health promotion** is defined as “the process of enabling people to increase control over, and to improve their health” **Disease prevention** refers to “specific, population-based and individual-based interventions for primary and secondary (early detection) prevention, aiming to minimize the burden of diseases and associated risk factors” **4. Can you tell me about the role/activities of community pharmacists in providing health promotion and disease prevention services?** • For example, chronic disease management, nutrition and physical activity, smoking cessation, oral health, vitamin and herbal products, weight management, and pediatric-related issues. **5. Based on the definitions I provided, could you give me examples of services you have offered to patients in your pharmacy related to health promotion and disease prevention?** **6. Please tell me about the resources that already exist within the pharmacy to promote health and prevent disease?** • For example, a consultation area, weighing scale, automated blood pressure measuring device, glucometer, brochures. • Why do you think these resources promote health and wellbeing? **7. Are patients followed up after receiving health promotion and disease prevention services?** • If yes—how are they followed up? **8. Do you think that promoting health and preventing disease are part of pharmacist’s professional responsibility?** • Why do you think so? **9. How does providing health promotion and disease prevention services affect healthcare delivery?** • Benefits: changes in team roles and responsibilities, changes in workload, improve patient outcomes, identifying health issues, and making appropriate evidence-based product recommendations. • Any negative aspects? **10. In your opinion, what challenges do you face in providing health promotion and disease prevention services to patients?** • For example, lack of time, absence of standard guideline for the service, and lack of health education materials for consumers **11. Which factors do you think may improve the ability of pharmacies to provide better health promotion and disease prevention services?** • For example, additional staff for proper counseling, practice-specific pharmaceutical guidelines, and educational resources for health promotion and disease prevention • What are the potential outcomes of improving pharmacists’ ability to provide better health promotion and disease prevention services? **12. Is there anything else that you feel has not been covered? Is there anything else about the role of community pharmacists in health promotion and disease prevention that you want to talk to me about?**

Each interview was audio-recorded with participants’ permission. Interviews were conducted in Arabic and transcribed verbatim. Transcripts were subsequently translated into English and checked for accuracy by AHI and II, who are fluent in both Arabic and English. To ensure translation accuracy and preserve the meaning of participants’ responses, the translated transcripts were reviewed against the original Arabic transcripts. Any discrepancies or ambiguities were discussed and resolved through consensus. Transcripts were not returned to participants for comment or correction, and formal member checking was not undertaken. However, credibility was supported through reflexive and collaborative analytical procedures. In addition, no repeat interviews were conducted.

### Data management and analysis

All data were anonymized during transcription to ensure participant confidentiality. Each participant was given an anonymous code (e.g., CP1, CP2). Thematic analysis was conducted following Braun and Clarke’s six-step approach [34], which includes familiarization with the data, generation of initial codes, searching for themes, reviewing themes, defining and naming themes, and producing the final report. Transcripts were imported into NVivo® QSR 14 to facilitate systematic coding and data organization. Two researchers (AHI and TA) independently coded the transcripts and compared their coding decisions. Initial codes were generated inductively from the data and grouped into broader themes through iterative comparison and refinement. The developing themes were continuously reviewed against both the coded extracts and the complete dataset to ensure coherence, consistency, and clear distinction between themes. Any discrepancies in coding or interpretation were discussed within the research team until consensus was reached. Themes were further refined through ongoing discussions among the research team to ensure accurate interpretation and representation of the data.

In this study, data saturation was the main determinant of the sample size. Data collection and analysis were conducted concurrently, with newly generated codes continuously compared with existing codes throughout the analytical process. Saturation was considered achieved when no new themes, concepts, or meaningful insights emerged from subsequent interviews. Following the point at which no new information was identified, a final interview was conducted to confirm saturation. Reflexivity was maintained through documentation of researchers’ assumptions and analytic decisions throughout the study. Emerging interpretations were regularly discussed within the research team to critically examine potential biases and ensure that themes remained grounded in the data.

Following completion of the inductive thematic analysis, a secondary deductive analysis was undertaken to provide a theoretical interpretation of the findings. Two researchers reviewed the finalized themes (AHI and TA) and mapped them to the COM-B framework using the original COM-B definitions. Differences in mapping were discussed until consensus was reached.

### Ethical considerations

The study received ethical approval from the Institutional Review Board at Al-Zaytoonah University of Jordan (IRB #15/12/2024–2025). The research was conducted in accordance with the principles of the Declaration of Helsinki. Participation was voluntary and written informed consent was obtained prior to all interviews. Data confidentiality and participant anonymity were strictly maintained and all identifying information was removed from transcripts.

## Results

In this study, 15 CPs were interviewed, with an average interview duration of 40 minutes. Data saturation was reached after 14 interviews. One additional interview was conducted to confirm that no new themes or meaningful insights emerged, after which no further recruitment was undertaken. The majority were female (n = 10, 66.7%), with a mean age of 27.5 (±6.1) years, and had less than 5 years of experience in community pharmacy practice (n = 11, 73.3%). Participants worked an average of 8 hours per day (SD ± 2.2). Two-thirds (n = 10, 66.7%) were the only pharmacist on duty per shift and most worked in single independent pharmacies (n = 12, 80.0%). Six CPs were unable to take part in the study due to time constraints and workload pressures. The demographic characteristics of participants are presented in Table 2.

**Table 2:** Demographic characteristics of the participants (N = 15).

	N (%) or Mean (±SD)
**Gender**	
Female	10 (66.7%)
Male	5 (33.3%)
**Average age (years)**	27.5 (±6.1)
Y**ears of experience in community pharmacy**	
<5 years	11 (73.3%)
5-10 years	3 (20%)
>10 years	1 (6.7%)
**Working sector other than community pharmacy**	
Yes	8 (53.3%)
No	7 (46.7%)
**Average monthly financial return**	
<500 JD	8 (53.3%)
500-1000 JD	7 (46.7%)
>1000 JD	0 (0%)
**Educational level**	
Bachelor’s Degree	11 (73.3%)
Pharm D	3 (20%)
Master’s Degree	1 (6.7%)
**Employment status**	
Employee	12 (80%)
Owner	3 (20%)
**Average number of patients who visit the pharmacy per day±(SD)**	67 (±48.8)
**Pharmacists per shift**	
One	10 (66.7%)
Two	5 (33.3%)
**Average working hours per day**	8.0 (±2.2)
**Pharmacy Type**	
Single	12 (80%)
Chain	3 (20%)

### Main themes from thematic analysis

Thematic analysis revealed four main themes that emerged from interview data: diverse and evolving roles of CPs in HPDP, barriers to providing HPDP services, professional requirements and capacity building, and impact of providing HPDP services.

### Theme 1: Diverse and evolving roles of CPs in HPDP

This theme describes the range of HPDP activities undertaken by CPs and their evolving role in promoting health and preventing disease within community pharmacy practice.

CPs described diverse and evolving roles in HPDP and consistently linked these services to improved public health outcomes. Although they agreed that health promotion could lead to disease prevention, none provided a definition fully aligned with that of the WHO. Instead, their explanations reflected a general understanding of reducing disease risk rather than a comprehensive public health perspective:

“Improving the health of the patient to reduce the risk of developing certain diseases…Health promotion leads to disease prevention; the two terms are not identical” (CP3)

In practice, CPs reported engaging in a wide range of HPDP-related activities. The most common activities included recording vital signs, providing ongoing patient education, monitoring medication adherence, and offering lifestyle advice, such as exercise, a healthy diet, weight management, and smoking cessation, particularly for patients at risk of or newly diagnosed with chronic conditions:

“We monitor patients with diabetes and hypertension by regularly recording blood glucose and blood pressure readings to assess their adherence to prescribed medications and determine if medication adjustments are needed.” (CP1)

“We offer nicotine gum to smokers and instruct them on its correct use.” (CP4)

“When patients are newly diagnosed with diabetes, hypertension, or dyslipidemia, I make sure to teach them about non-pharmacological therapies that aid in disease control, such as healthy food and exercise.” (CP5)

Patient education also extended to addressing missed medication doses, correct medication use, potential side effects, and drug interactions:

“CPs play a major role in educating patients on how to deal with missed medication doses, especially those relating to hormones and contraceptives.” (CP3)

Beyond chronic disease management, many CPs reported advising on minor dermatological issues, dental care, and providing first aid for wounds, burns, and bruises:

“We examine the skin and treat existing problems using mixtures, moisturizers, and sunscreen.” (CP6)

“In the area of dental care, there are people complaining of gum sensitivity; we can advise them to use mouthwash, change the toothbrush regularly, and use a soft toothbrush for sensitive teeth and gums.” (CP10)

“Providing first aid for wounds, burns, and bruises” (CP14)

Several CPs also highlighted their expanded roles during the COVID-19 pandemic, including education about infection prevention, vaccine administration, and advice on supplements to support immunity:

“We [CPs] advise patients to take vitamins that boost immunity, and to use masks as a means of prevention, and to receive annual flu vaccine, especially for people with diseases or those with weak immunity.” (CP5)

“During the Coronavirus pandemic, we [CPs] had a major role in giving the vaccine.” (CP11)

### Theme 2: Barriers to providing HPDP services

This theme focuses on the factors that hinder or limit the effective provision of HPDP services in community pharmacy settings.

CPs described multiple barriers that limited their capacity to provide HPDP services effectively. Many CPs identified excessive workload as a primary barrier to delivering these services. Often CPs found themselves incapable of providing thorough patient engagement due to the high volume of patients relative to the limited number of pharmacists available during each shift (i.e., often only one pharmacist per shift). Consequently, participants emphasized the necessity for more than one pharmacist per shift to manage workload and ensure the provision of HPDP services without compromising quality of care:

“The average number of patients in our pharmacy is approximately 100 per day. We [CPs] provide daily services for at least half of them with an average time ranging from 5 minutes to one hour depending on the patient, the number of patients present and waiting, and whether there is another pharmacist in the pharmacy… it [HPDP service] requires time and effort from the pharmacist” (CP4)

“If I spend a long time educating one patient, there will be two or three patients behind waiting for their turn. Therefore, it is very important to have more than one pharmacist in the pharmacy per shift, especially in busy pharmacies.” (CP9)

Patient-related factors were also described as barriers. Several CPs indicated a lack of willingness among some patients to utilize HPDP services, particularly when these services required additional time or were perceived as intrusive. Lack of patient interest and trust in pharmacists’ advice were noted as challenges to the implementation of preventive health interventions:

“There are people who don’t care about the advice…they would say: I didn’t ask you, why are you telling me?... and they may get annoyed by the advice. Some people don’t have trust the advice.” (CP6)

Organizational barriers were also highlighted. Many CPs described a lack of support, motivation, and appreciation from pharmacy owners, which diminished their enthusiasm for offering HPDP services. A number of CPs indicated that pharmacy owners prioritized profit over the quality or extent of preventive services:

“The appreciation and respect for the pharmacist from the pharmacy owner is very important, and it becomes a motivation for him to give more. If there is no appreciation for the pharmacist, his enthusiasm fades.” (CP9)

“The pharmacy owner has a huge role, especially if there is no appreciation and he is only concerned with the profit side.” (CP10)

Financial considerations were another important barrier. More than half of the CPs emphasized the necessity for financial incentives to support HPDP service provision. These services are generally provided free of charge in Jordan, which affects both the willingness and capacity of CPs to offer them. On the other hand, some CPs expressed concern that introducing fees could discourage patients from utilizing HPDP services, as many already seek CPs’ help because no consultation fees are required:

“The service provided would be for a fee, and in this case, pharmacists will start to compete in providing the service” (CP1)

“There is no financial compensation for this service…but if we force the patient to pay for the services provided by the pharmacist, the patient will withdraw and stop coming to us in the first place…patient comes to us because he doesn’t have the money for the physician’s consultation, he doesn’t have the money to pay it.” (CP4)

### Theme 3: Professional requirements and capacity building

This theme focuses on the resources, competencies, and support mechanisms perceived as necessary to strengthen pharmacists’ capacity to provide HPDP services. In contrast to the barriers identified in Theme 2, participants discussed factors that could facilitate and enhance the delivery of HPDP services in community pharmacy practice.

Many CPs mentioned that the availability of a private counseling room, or at least a consultation area within the pharmacy, would substantially facilitate confidential patient discussions and enhance patient engagement during the delivery of HPDP services:

“I wish we had a dedicated space for providing pharmaceutical counseling.” (CP1)

In addition, physical comfort was highlighted as important. Participants reported that patients prefer pharmacies with spacious, air-conditioned interiors, ready access to first-aid supplies, and on-site parking facilities:

“The size of the pharmacy can also affect the comfort of the visitor; a large and air-conditioned pharmacy is better for the visitor.” (CP2)

“The presence of parking spots outside in front of the pharmacy… so that no one tells you they [Patients] are in a hurry.” (CP8)

Patient receptiveness to HPDP services was reported to be closely related to their level of awareness and health literacy. Many CPs emphasized the importance of using visual aids within the pharmacy (e.g., screens, brochures, and informational stickers) to educate patients about diseases and nutritional supplements. Moreover, the use of social media platforms and the organization of free medical or nutritional awareness days were highlighted as strategies to increase patient awareness:

“Honestly, people’s response to pharmaceutical counseling differs from one area to another depending on individuals’ culture and awareness...a medical day in the pharmacy, where there is a physician or a skincare specialist, for example, to provide certain services to patients or visitors.” (CP5)

“There must continuously be external awareness activities for pharmacists in malls, medical care centers, and in every suitable place, with the aim of educating people, promoting their health, and preventing them from diseases.” (CP12)

“Stands for a certain medication like Panadol or iron products; it’s normal to place them in the pharmacy…A WhatsApp group for the pharmacy so any product from dietary supplements or a dermatological product that’s not classified as a medicine, we send it to the group… We distribute brochures provided by pharmaceutical companies.” (CP15)

Nearly all CPs indicated that employing telecommunication tools (e.g., telephones, WhatsApp, Wi-Fi access) and basic medical monitoring devices (e.g., blood pressure monitors and glucometers) was essential to effectively track patient outcomes and enhance adherence. Furthermore, they mentioned that they followed their patients either through regular in-pharmacy visits or remotely via phone calls or WhatsApp messaging:

“I follow up with patients through the phone, either by calls or WhatsApp…I also take feedback from them when they return to the pharmacy and check on the results of the treatment, the advice, or the supplement they took from me during their last visit.” (CP8)

“I always tell them: ‘come back and let us know what happens with you’. If we asked them for tests, we tell them: ‘come back and show us the results’…We can also follow up with them through WhatsApp. The availability of resources like a blood pressure monitor and a glucose meter helps in providing the service.” (CP12)

CPs consistently considered close collaboration with physicians to be vital for optimizing patient care, including making referrals when needed and coordinating medication adjustments. All CPs also agreed that providing HPDP services constituted a fundamental aspect of their professional responsibility:

“Also, patients who suffer from hypothyroidism; we can, in collaboration with the physician, adjust the dose of levothyroxine... referring medical cases to the appropriate specialist according to the condition” (CP3)

“It [providing HPDP services] is part of the pharmacist’s responsibility and duty… we are the link between the physician and the people…we are the ones the patient interacts with the most; the patient comes, stands behind the counter, and asks all the questions, and we answer. This service is not provided by anyone else, not the physician, not the nurse, nor the lab technician.” (CP7)

Many CPs further emphasized that pharmacists must possess confidence and intrinsic motivation to deliver HPDP services effectively:

“The pharmacist must have confidence in himself and his knowledge so that the listener trusts him and takes his advice.” (CP2)

“If the pharmacist himself doesn’t want to provide the service, you can’t force him to give it…the pharmacist must want to give it willingly.” (CP5)

Most CPs stated that adequate knowledge and skills should be acquired during undergraduate pharmacy education prior to graduation. In addition, they highlighted the importance of ongoing access to continuous professional development programs and up-to-date educational resources after graduation.

However, many CPs reported that they preferred to refer special populations (e.g., psychiatric patients, cancer patients, pregnant women, breastfeeding mothers, pediatric and elderly patients) and individuals with dermatological conditions to physicians, instead of providing HPDP services themselves, primarily due to insufficient training and knowledge in these areas:

“Lack of knowledge; the pharmacist doesn’t have information to provide…regarding the group of breastfeeding and pregnant women, pharmacists do not have enough information about this group, the safe medications, and how to deal with complications of breastfeeding and pregnancy…” (CP3)

“The pharmacist must be educated regarding skin conditions and know how to treat the related problems”. (CP6)

“Highlighting this concept (HPDP services) and its method of application during the university years... how to be a pharmacist upon graduation and not just a seller…. regular training courses for pharmacists to increase their experience so they can develop their knowledge in line with updated information.” (CP15)

### Theme 4: Impact of providing HPDP services

This theme describes the perceived benefits of HPDP services for patients, pharmacists, pharmacies, and the wider healthcare system.

The provision of HPDP services by CPs was perceived to yield numerous benefits for patients, pharmacists, pharmacies, and the healthcare system. Many CPs observed that HPDP services contributed to a reduction in the prevalence of chronic diseases and, ultimately, could decrease hospitalizations, morbidity, mortality, and healthcare costs:

“It reduces chronic diseases…reduces the pressure on hospitals and health centers…reduces the financial cost of treatment for the patient.” (CP15)

Additionally, CPs emphasized that providing HPDP services could lead to improved patient outcomes, including quality of life and patient adherence:

“For the patient, their quality of life becomes better.” (CP1)

“Improving patients’ health, increasing disease control, enhancing patients’ adherence to their medications, and avoiding disease complications and medication side effects.” (CP13)

Providing HPDP services was also reported to have a positive impact on pharmacists themselves, including professional growth, increased job satisfaction, and greater respect and credibility within the community. CPs described gaining valuable experience and motivation for lifelong learning through their HPDP activities:

“At the pharmacist’s level, he benefits from the information he applies, gains experience, and this service can motivate him to read more and broaden his horizons. We change society’s perception and convey the idea that the pharmacist is not just a seller.” (CP1)

“Credibility and building trust with the patient, and they come back and ask again.” (CP7)

“The market value of the pharmacist increases…trust, value, and respect for the pharmacist will increase” (CP14)

Sustained HPDP services were also recognized as drivers of positive change at the pharmacy level. CPs reported improvements in the pharmacy’s reputation within the community, stronger patient loyalty, and indirect increases in financial performance attributed to higher customer satisfaction:

“The pharmacy’s reputation becomes good among people in the community…the good reputation of the pharmacy spreads because they have a skilled pharmacist who advises patients properly, and as a result, patients improve and sales in the pharmacy increase.” (CP1)

“Increasing trust in the pharmacist, and thus the pharmacist gains customers...increasing financial income” (CP4)

### Deductive mapping of themes to the COM-B framework

**Fig 1:**
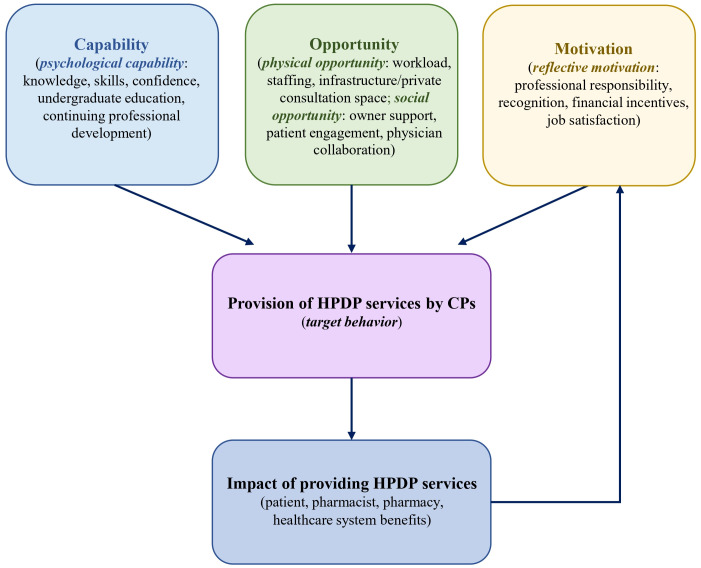
Conceptual model of factors influencing CPs’ provision of HPDP services, adapted from the COM-B model.

To provide a theoretical interpretation of the findings, the identified themes were deductively mapped to the COM-B framework (Table 3). This mapping suggested that CPs’ provision of HPDP services was influenced by all three COM-B constructs. More specifically, the identified factors were primarily associated with psychological capability, physical and social opportunity, and reflective motivation, whereas physical capability and automatic motivation were not strongly represented in participants’ accounts. Capability-related factors included pharmacists’ knowledge, skills, confidence, undergraduate education, and continuing professional development. Opportunity-related factors included workload, staffing, infrastructure, private consultation space, owner support, patient engagement, and collaboration with physicians. Motivation-related factors included professional responsibility, professional recognition, financial incentives, and job satisfaction. Together, these factors influenced CPs’ engagement in HPDP services. In addition, the impacts associated with HPDP service provision, including benefits for patients, pharmacists, pharmacies, and the healthcare system, may reinforce pharmacists’ motivation to continue engaging in these activities. Fig 1 provides a conceptual representation of the relationships between the identified COM-B constructs, HPDP service provision, and the impacts associated with service delivery.

**Table 3:** Deductive mapping of study themes to the COM-B framework.

Study theme	COM-B construct
Diverse and evolving roles of CPs in HPDP	Behavior
Barriers to providing HPDP services	Opportunity, Motivation
Professional requirements and capacity building	Capability, Opportunity
Impact of providing HPDP services	Motivation

## Discussion

This qualitative study used thematic analysis to investigate Jordanian CPs’ perspectives on providing HPDP services. Exploring the experiences and perspectives of CPs is crucial for understanding their current role, recognizing existing practices, identifying barriers, and uncovering opportunities to improve the quality and effectiveness of HPDP within community pharmacy settings in Jordan.

In this study, the diverse range of activities reported by participating CPs highlights their expanding contribution to HPDP, consistent with findings from previous studies [23,35]. Participating CPs described involvement in a wide variety of HPDP activities such as chronic disease management, patient education, promotion of healthy lifestyles, and support for medication adherence. These findings are consistent with a systematic review identifying cardiovascular disease prevention, hypertension and diabetes management, and smoking cessation as primary HPDP services provided by CPs across the United Kingdom, United States, Australia/New Zealand, Europe, and Canada [36]. However, service profiles appear to vary across different contexts. For example, Ethiopian CPs mainly offer family planning advice and interventions targeting substance and alcohol misuse [37,38]. This variation suggests that the scope of HPDP services may be influenced by local cultural practices and healthcare system structures.

Beyond describing a broad range of activities, our findings also revealed an important conceptual dimension. Although CPs recognized the link between health promotion and disease prevention, their explanations primarily emphasized individual patient counseling and disease risk reduction rather than a broader public health framework. This indicates that although pharmacists are increasingly involved in public health activities, their understanding of health promotion remains largely focused on individual patient care rather than broader population-level strategies. Similar observations have been reported in previous studies, where pharmacists conceptualized health promotion mainly as behavior change advice and clinical risk management delivered through individual patient encounters [39,40]. The literature further suggests that this pattern may reflect the traditionally clinical and patient-centered orientation of community pharmacy practice, with ongoing calls for stronger public/population health training and systems support to enable broader contributions [12,41].

Our study also revealed several interrelated barriers to the effective delivery of HPDP services, including high patient volumes, limited staffing, and inadequate time, as well as patient-related factors such as limited interest or trust in preventive advice, insufficient appreciation from pharmacy owners, and the absence of financial incentives. These findings are consistent with previous studies conducted in Spain, Saudi Arabia, Canada, and Georgia, which similarly highlighted staff shortages, time constraints, and inadequate financial support as main challenges faced by CPs [8,42–44]. Furthermore, the literature consistently indicates that insufficient staffing exacerbates workload pressures, leading to increased job stress and diminished satisfaction among pharmacists, which may ultimately compromise the quality of care provided [45–47]. Evidence from studies conducted in other Middle Eastern and low- and middle-income settings also suggests that workforce shortages, limited infrastructure, insufficient remuneration, and restricted interprofessional collaboration are common challenges affecting pharmacists’ ability to provide public health services [12,43,46,48]. Similar barriers have also been identified in qualitative research conducted in comparable healthcare settings [49]. Collectively, these findings suggest that many of the challenges identified in our study extend beyond the Jordanian context and reflect broader health system and organizational factors that influence pharmacists’ engagement in HPDP services. Therefore, addressing these barriers through targeted policy reform and appropriate resource allocation may enhance CPs’ engagement in HPDP.

Regarding professional requirements, this study highlighted the importance of environmental factors, such as private counseling or consultation areas to support the delivery of HPDP services. Similar research from Saudi Arabia indicated that limited private counseling spaces significantly restricted pharmacists’ ability to deliver cardiovascular health promotion services effectively [43]. Furthermore, Atif et al. emphasized the importance of dedicated counseling areas for weight management interventions, reflecting broader international agreement on the necessity of suitable physical spaces for effective HPDP implementation [49].

In addition, our study underscores the necessity of clearly defining CPs’ roles and formally recognizing their contributions to public health. Participating CPs noted that patient awareness and health literacy significantly influence service uptake. This aligns with previous literature noting limited public understanding of pharmacists’ broader healthcare roles as a critical barrier [42]. Social media and community outreach activities may serve as valuable tools to enhance public perception and increase engagement with pharmacy services [50,51]. Additionally, participating CPs emphasized the importance of telecommunication and digital technologies for patient follow-up and monitoring, consistent with findings from previous studies identifying technologies and social media as important considerations for pharmacists’ public health role [52].

Collaboration with physicians emerged as essential for optimizing patient care, particularly through referrals and coordinated treatment adjustments. Previous research has similarly identified limited coordination with physicians as a barrier to pharmacists’ involvement in HPDP [8,42,45,46,48,53]. Structured interprofessional workshops and regular joint clinical activities between pharmacists and physicians may strengthen collaboration and enhance integrated service delivery [54].

Training emerged as a critical factor influencing pharmacists’ confidence and competence in delivering HPDP services. A study in Saudi Arabia reported gaps in pharmacists’ knowledge, highlighting the need for further training and professional development [55]. Strengthening pharmacy curricula to emphasize practical skills and up-to-date knowledge, particularly for special populations, is strongly recommended. National initiatives such as continuing professional development programs provide opportunities to integrate dedicated HPDP modules into mandatory training hours [56].

Participants perceived that pharmacist-led HPDP services could improve patient outcomes, enhance medication adherence, strengthen patient-pharmacist relationships, and potentially reduce healthcare costs and healthcare system burden. These perceptions are consistent with findings reported in previous studies. For example, a study from Selangor reported that CPs believed pharmacists’ HPDP interventions could improve community health outcomes [57]. Further, pharmacists’ active involvement in chronic disease management has been shown to improve patient access to care, maintain patient safety, and enhance quality of life [58].

To provide a broader conceptual interpretation of the findings, the identified themes were deductively mapped to the COM-B framework. This approach, which combines inductive thematic analysis with subsequent deductive mapping, has been used in previous qualitative health research to facilitate theory-informed interpretation of findings [59–61]. The COM-B mapping suggests that pharmacists’ engagement in HPDP services is influenced by the interaction of capability-, opportunity-, and motivation-related factors rather than any single barrier or facilitator in isolation. The findings indicate that enhancing pharmacists’ involvement in HPDP services requires not only improving knowledge and skills but also addressing organizational, environmental, and motivational influences. For example, educational interventions may increase capability, but their effectiveness is likely to be limited if pharmacists continue to face workload pressures, inadequate staffing, limited consultation facilities, or insufficient organizational support. Similarly, strengthening motivational drivers such as professional recognition and job satisfaction may encourage sustained engagement in HPDP activities.

The findings of this study have several implications for policy and practice. At the educational level, HPDP competencies should be further integrated into undergraduate pharmacy curricula and continuing professional development programs. At the organizational level, community pharmacies should be supported in establishing private consultation areas and allocating sufficient resources for preventive care activities. At the health system level, stronger collaboration between pharmacists and physicians should be encouraged through structured referral pathways and interprofessional initiatives. Pharmacist-led HPDP services could also be more formally integrated into national public health strategies, particularly those targeting noncommunicable disease prevention, smoking cessation, vaccination, and health screening programs. Public awareness campaigns may also help improve understanding of pharmacists’ public health roles and increase patient engagement with HPDP services. Consideration should also be given to developing reimbursement or incentive mechanisms to support the sustainable delivery of HPDP services within community pharmacies.

### Strengths and limitations

The qualitative design of this study provided an in-depth understanding of CPs’ perspectives regarding the provision of HPDP services. The presence of one interviewer and the use of the interview topic guide provided standardization in the depth and breadth of topics covered and minimized variation in the way interviews were conducted. Despite these strengths, several limitations should be acknowledged. First, most participants were recruited through snowball sampling, which may have introduced selection bias. Despite this, given the workload and limited availability of Jordanian CPs, this target population is not easily accessible for research, which may decrease their willingness to participate. Furthermore, participants were recruited from the capital, Amman, which increases the potential for selection bias and may limit the generalizability of the findings to CPs practicing in other regions in Jordan, although efforts were made to ensure diversity in terms of participants’ characteristics. However, the National Human Resources for Health Observatory Annual Report indicates that the highest ratio of pharmacists per 10,000 population is found in Amman [62]. Furthermore, the perspectives of CPs in this study are broadly similar to those reported in the literature, which may support the transferability of the findings. In addition, most participants were relatively young and had fewer years of professional experience. As a result, the findings may primarily reflect the perspectives of early-career pharmacists, whose experiences, expectations, and attitudes toward HPDP services may differ from those of more experienced practitioners. Therefore, caution should be exercised when interpreting the findings and transferring them to the wider population of CPs in Jordan.

In addition, interviews were conducted in Arabic and translated into English, which may have resulted in the loss of subtle linguistic nuances. However, efforts were made to minimize this risk by reviewing translated transcripts against the original Arabic transcripts and resolving any discrepancies through discussion among the research team. Furthermore, as with all interview-based studies, the possibility of social desirability bias cannot be excluded, as participants may have presented their professional roles and practices more favorably during the interviews.

Interviewer bias refers to systematic differences in how information is solicited, recorded, or interpreted. It is important to note that the potential for interviewer bias was minimized, as the researcher (II) who conducted the interviews was a full-time lecturer and did not practice in community pharmacy. Also, she took care to avoid expressing personal opinions during data collection by using a reflexive approach. To further support confirmability, the researcher maintained an audit trail of all records resulting from the study, including audio recordings, transcripts, field notes, findings, and detailed descriptions of the analysis.

### Quality criteria for this research

A number of strategies were employed to establish trustworthiness in this study. To ensure credibility, the two researchers (AHI and TA) had previous experience conducting qualitative studies and were familiar with the methodological approach used in this research. Moreover, the interviewer (II) was trained in qualitative research methodology prior to beginning the study. Independent coding of transcripts by two researchers, followed by consensus discussions to finalize themes (investigator triangulation), was conducted. Additionally, verbatim quotations were used in reporting findings to support interpretations. Finally, prolonged engagement with the data was achieved through repeated reading and immersion during analysis. Regular discussions among the research team were undertaken throughout the analytical process to critically examine emerging interpretations and enhance the credibility of the findings.

To enhance transferability, a clear description of the study setting, participant characteristics, and inclusion criteria was provided. Also, a detailed explanation of purposive and snowball sampling strategies was included. To allow comparison with other contexts, transparent reporting of data collection and thematic analysis procedures was undertaken.

To improve dependability, systematic documentation of the research process, including methodological and analytical decisions, was undertaken. Detailed records of interviews, field notes, and coding decisions were maintained as part of an audit trail. To ensure confirmability, reflexive practice was adopted throughout both data collection and analysis. Researchers continuously reflected on their assumptions and interpretations and discussed these within the research team to minimize the influence of personal biases on the findings. Transparent reporting of findings in accordance with the Consolidated Criteria for Reporting Qualitative Research (COREQ) checklist was also undertaken.

## Conclusions

This qualitative study explored the perspectives of CPs regarding the provision of HPDP services in Jordan. The findings indicate that CPs play an active role in patient education, medication adherence support, and chronic disease management. However, the provision of HPDP services remains constrained by barriers such as high workload, lack of time, insufficient staffing per shift, limited appreciation and support from pharmacy owners, absence of financial incentives, and patient-related factors such as limited awareness or trust. Addressing these barriers is essential to strengthen the contribution of CPs to preventive care. HPDP services were perceived by participants to enhance clinical outcomes, support patients’ long-term health, and strengthen trust between pharmacists and patients. Participants also reported that these services improved professional satisfaction and enhanced pharmacy reputation, with potential indirect financial benefits. These findings provide valuable insights for policymakers, educators, and healthcare stakeholders in Jordan.

The study highlights the need for training and professional development to equip CPs with the competencies required for effective HPDP service delivery. In addition, efforts should focus on improving infrastructure within community pharmacies, including the provision of private consultation areas, strengthening collaboration between pharmacists and other HCPs, and developing supportive policies that facilitate the sustainable delivery of HPDP services. Future research should explore strategies to enhance CP engagement in HPDP services and evaluate the outcomes of training interventions and pharmacist-led preventive services. In addition, future studies should examine the perspectives of patients, physicians, and policymakers regarding the implementation of HPDP services within community pharmacy settings.

## Supporting information

S1 File Completed COREQ checklist. (PDF)
